# Postoperative Delirium and Postoperative Cognitive Dysfunction in Patients with Elective Hip or Knee Arthroplasty: A Narrative Review of the Literature

**DOI:** 10.3390/life12020314

**Published:** 2022-02-20

**Authors:** Petros Kitsis, Theopisti Zisimou, Ioannis Gkiatas, Ioannis Kostas-Agnantis, Ioannis Gelalis, Anastasios Korompilias, Emilios Pakos

**Affiliations:** 1 Department of Orthopaedic Surgery, School of Medicine, University of Ioannina, Stavrou Niarchou Street, 45500 Ioannina, Greece; th.zisimou@hotmail.com (T.Z.); igkiatas@uoi.gr (I.G.); ioanniskostas@hotmail.com (I.K.-A.); idgelalis@gmail.com (I.G.); koroban1960@gmail.com (A.K.); epakos@yahoo.gr (E.P.); 2 Orthopaedic Department, Nicosia General Hospital, Lemesou 215, Strovolos, 2029 Nicosia, Cyprus; 3 Hippocrateon Hospital, 6-12 Psaron Street, Engomi, 2408 Nicosia, Cyprus

**Keywords:** elective total knee replacement, elective total hip replacement, delirium, cognitive dysfunction, neurobehavioral manifestations, mental disorder

## Abstract

Postoperative delirium (POD) and postoperative cognitive dysfunction (POCD) are common complications following total knee arthroplasty (TKA) and total hip arthroplasty (THA), affecting the length of hospital stay and increasing medical complications. Although many papers have been published on both conditions in this setting, no reviews have currently been written. Thus, the purpose of our study is to summarize the current literature and provide information about POD and POCD following elective THA or TKA. Our literature search was conducted in the electronic databases PubMed and the Cochrane library. We found that POD is a common complication following elective THA or TKA, with a median incidence of 14.8%. Major risk factors include older age, cognitive impairment, dementia, preoperative (pre-op) comorbidities, substance abuse, and surgery for fracture. Diagnosis can be achieved using tools such as the confusion assessment method (CAM), which is sensitive, specific, reliable, and easy to use, for the identification of POD. Treatment consists of risk stratification and the implementation of a multiple component prevention protocol. POCD has a median incidence of 19.3% at 1 week, and 10% at 3 months. Risk factors include older age, high BMI, and cognitive impairment. Treatment consists of reversing risk factors and implementing protocols in order to preserve physiological stability. POD and POCD are common and preventable complications following TKA and THA. Risk stratification and specific interventions can lower the incidence of both syndromes. Every physician involved in the care of such patients should be informed on every aspect of these conditions in order to provide the best care for their patients.

## 1. Introduction

Total hip and total knee replacements are among the most commonly performed surgical procedures at present. These procedures are two of the most cost-effective and consistently successful surgeries performed in orthopedics. The most common indication for both surgeries is osteoarthritis. Total hip arthroplasty (THA) and total knee arthroplasty (TKA) provide reliable outcomes, such us pain relief, functional restoration, and overall improved quality of life. Although the benefits outweigh the drawbacks, these procedures do not come without complications. Delirium and postoperative cognitive dysfunction are such, with their numbers increasing due to the increasing number of THAs and TKAs performed. Every physician involved in the care of postoperative (post-op) THA and TKA patients should be informed on every aspect of these conditions in order to provide the best care for their patients [1,2,3,4,5,6].

Delirium is a neuropsychological syndrome characterized by a disturbance in attention and awareness, which is an acute change from baseline, disturbance in cognition and fluctuating in severity during the course of the day. Postoperative delirium (POD) is a specific subset of delirium that is not related to emergence from anesthesia. Postoperative cognitive dysfunction (POCD) is associated with increased mortality, risk of leaving the labor market prematurely, and a dependency on social transfer payments. It is a syndrome characterized by the impairment of cognitive function that is distinct from delirium and dementia. Features of POCD include limitations in memory, intellectual ability, and executive function. Both syndromes are common complications following surgery, and are associated with prolonged hospital stay [7,8,9,10], medical complications [7,11,12,13], and delayed mobilization [10].

Nonetheless, there is a deficiency of written reviews of the literature regarding POCD and POD following elective TKA or THA. Therefore, the main goal of this review is to summarize the literature and provide the reader with the necessary information regarding these entities. More specifically, it will provide information on the epidemiology, incidence, risk factors, diagnosis, and treatment of both syndromes in patients undergoing these procedures, in order to make the reader more familiar with these entities and to enhance the knowledge in the clinical practice, as these syndromes are in most cases preventable.

## 2. Materials and Methods

**Scheme 1:**
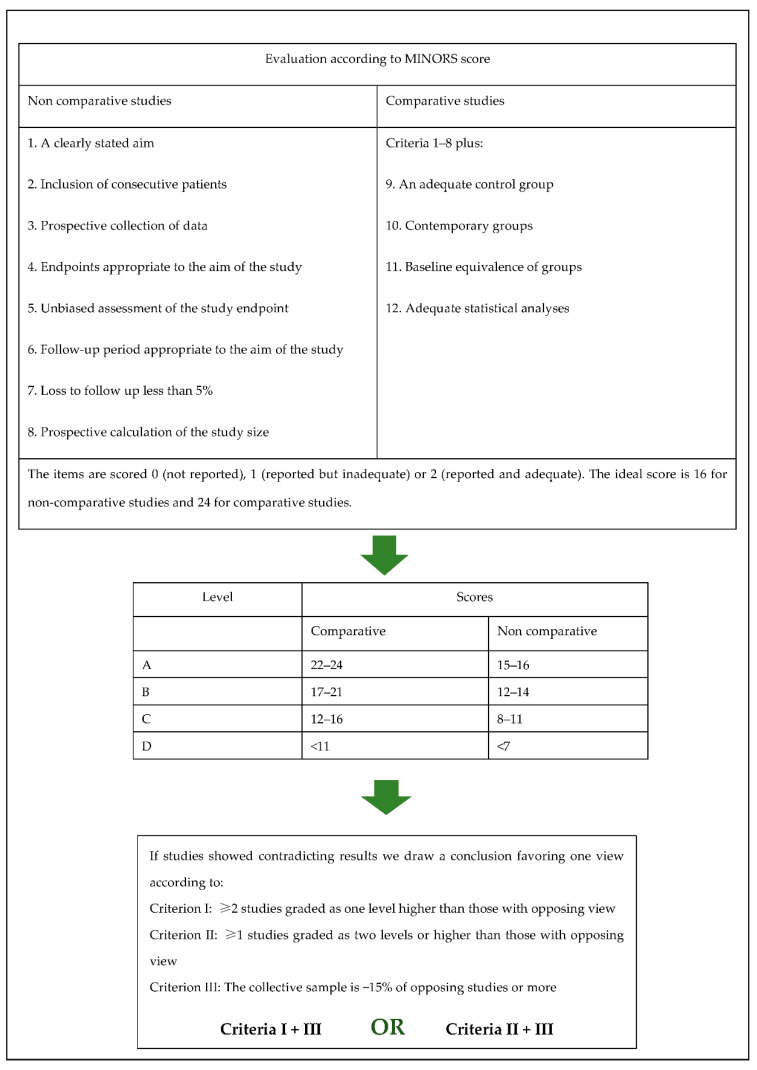
Qualitative evaluation of studies with MINORS scores and criteria used when contradicting evidence were present.

We searched the electronic databases PubMed (in mesh terms) and the Cochrane library, using the following terms: “mental disorder”, “neurobehavioral manifestations”, “elective hip arthroplasty”, and “elective knee arthroplasty”. We set no date of publication limitation. Our search resulted in over 560 articles, from which less than one hundred fit the inclusion criteria. After that, we performed a manual search of the references in the selected articles. We consequently obtained over 95 articles that were included in our study. The inclusion criteria for our review were: articles in the English language, articles related to elective hip replacement or elective knee arthroplasty, articles related to POD or POCD, and, more specifically, articles that referred to POD or POCD as postoperative complications. Case reports were excluded. Studies were qualitative assessed using the methodological index for non-randomized studies (MINORS), as explained in Scheme 1 [14]. Following that, we divided studies according to their score into four levels, A, B, C, and D. Where contradicting evidence were present we used the following criteria in order to draw a conclusion favoring the one view vs. the other: criterion I was fulfilled if two or more studies were graded as one level higher than those of the opposing view, criterion II was fulfilled if one or more studies were graded as two levels higher, and criterion III (sample criterion) was fulfilled if the collective sample was −15% of opposing studies, or more. To draw a conclusion favoring one view, we required either criterion I plus criterion III, or criterion II plus criterion III, otherwise we were unable to make a concluding statement (Scheme 1).

## 3. Results and Discussion

### 3.1. POD

Delirium is defined by the Diagnostic and Statistical Manual of the American Psychiatric Association (DSM-5) as a neuropsychological syndrome characterized by disturbance in attention and awareness (which is an acute change from baseline, fluctuating in severity during the course of the day), and an additional disturbance in cognition, not explained by a pre-existing or evolving neurocognitive disorder or a reduced level of arousal. Lastly, there should be evidence of an attributable cause. POD is a specific subset of delirium, and is not related to emergence from anesthesia [15]. It usually occurs within the first 72 h post-op [16,17,18,19,20,21], with most cases occurring between 24–48 h [16,17,18,20,21]. On average, POD lasts for 1.8 days [22].

#### 3.1.1. Epidemiology

There was a considerable variation among the incidences reported by individual studies (0–48%), with a median of 14.8%. Most of the studies fell between 10–15% (Table 1) [9,11,12,13,16,18,19,20,21,22,23,24,25,26,27,28,29,30,31,32,33,34,35,36,37,38,39,40,41,42,43,44,45,46].

There are numerous factors responsible for the great variation in the literature. Firstly, the post-op day in which researchers evaluated the presence of POD differed between the studies. In a meta-analysis, JE Scott et al. reported that the incidence was higher among the studies that evaluated the presence of POD in the first 48 h post-op than studies that conducted the evaluation >72 h post-op [21]. Furthermore, different researchers used different tools/criteria to diagnose POD; each diagnostic tool possesses different levels of sensitivity and specificity, and this is reflected in the incidence range reported above. In some studies, the evaluation took place by psychiatrists, whereas in other studies evaluation was carried out by an orthopedic surgeon, or by the ward nurses in some cases. Additionally, the difference in the inclusion and exclusion criteria between the studies is a major confounding factor. Considering the above, the need for homogenous research regarding the incidence of POD is clear.

#### 3.1.2. Risk Factors

Identifying the risk factors for POD after elective THA/TKA is of the utmost importance since the basis for treatment is determined by risk stratification protocols; thus, an accurate presentation of the risk factors helps the reader to understand the treatment approach, which will be discussed later, and also sets the basis for future risk stratification protocols regarding this specific subset of patients (Table 2).

Older age (>70 years old) is clearly correlated with increased risk of POD [9,10,11,12,17,18,31,47,48,49,50,51]. Some reports show that for every year of age the risk of POD increases by a factor of 1.1–1.15 [18,47]. Studies addressing the relationship between sex and POD show contradictory results, with some suggesting that males have a higher risk of developing POD [7,12,50,52], and others that implying there is no association [8,10,11,18]. Thus, we cannot make a key statement regarding sex and POD. There is also a lack of agreement in the literature regarding the race factor, with some stating that the white race has a higher risk of POD than other races [7], whereas others found no correlation [16].

Preoperative cognitive decline increases the risk of POD [4,11,25,31,32,37,43,50]; however, the existing literature failed to clearly correlate a cognitive function screening test, i.e., mini-mental state examination (MMSE), and the risk of POD, with few studies showing a positive interconnection between ΜΜSE and POD [11,16,20,37], while others show no statistically significant interrelationship [31,32,43,50]. In summary, more research is needed to determine which cognitive function screening tool correlates with the POD risk. Regarding dementia, the literature shows a statistically significant correlation with POD, with one study reporting an OR (odds ratio) of 10.4 [10,18]. It is worth noting that there are only a few studies addressing this relationship, because the majority of authors exclude demented patients from their studies.

Preoperative (pre-op) comorbidities, such as electrolytic imbalances, abnormal weight loss, cardiac disease (congestive heart failure, arrhythmias, coronary disease), coagulopathy, diabetes, asthma, chronic obstructive pulmonary disease (COPD), stroke, smoking, renal disease, and ASA grade 3 or higher (American Society of Anesthesiologist physical status grade) correlates with POD [7,9,10,17,47,50,51]. However, a number of questions regarding pre-op comorbidity remain to be addressed. In each individual study, the authors examined a specific subset of comorbidities, leaving others out. Furthermore, some authors addressed comorbidities via a score, such as ASA or the Charlson comorbidity index (CCI). Considering the above, we are unable to conclude how specific diseases correlate with POD, although we can state with certainty that patients with comorbidities are more likely to develop POD than patients without.

Obstructive sleep apnea (OSA) seems to correlate with POD [16,53]. R M Gupta et al. stated that OSA is not correlated with POD; however, there are some important limitations to their study. More specifically, since theirs was a case–control study, it is susceptible to bias, and there was no clear statement of the method used for the diagnosis of POD (this was noted by caregivers) [54].

The type of surgery (TKA or THA) and POD seems to be correlated, with some studies showing a stronger relationship between TKA and POD than THA [17,31,47]. Moreover, a meta-analysis by J E Scott et al. showed the same result regarding the type of surgery and POD, although without statistical significance [21]. Additionally, if we compare the fracture vs. elective surgeries, the first pose a greater risk of the development of POD than the latter [21]. Other than the type of surgery itself, increased blood loss during surgery correlates with POD [10,31], although the number of blood transfusions does not correlate with POD [10]. It is worth mentioning that in non-elective settings, operation duration of >3 h significantly increases the risk of POD by six times compared to a duration of <3 h [55]. Researching the literature for elective THA/TKA, no studies were found that addressed this matter with statistically significant results.

#### 3.1.3. History of Neuropsychiatric Conditions

Regarding depression and POD, there is contradicting evidence in the literature. Although some authors referred to the correlation as an axiom, reviewing previous papers showed that some studies indicate an increased risk of POD in patients with depression [7,11,52], while others found no connection [20,37,47,56]. Schizophrenia seems to correlate with POD; for example, J Joseph Gholson et al. reported a positive interconnection between these factors, with an OR of 11.2 [57]. Parkinson’s disease (PD) increases the incidence of POD. For instance, Jared M Newman et al. reported an OR of 2.61 in patients with PD vs. control group [58,59]. Even though numerous papers have addressed the relationship between alcohol abuse and POD, we are still not able to make a clear statement due to the contradicting results. Some authors, such as Keith T Aziz et al., suggest a strong relationship between alcohol abuse and POD [7,38], while others report no relationship [11,37,47,52]. In addition, we can assume that studies that addressed pre-op comorbidities with ASA scores included patients with alcohol dependence or abuse, so it is difficult to make a key statement. Since alcohol dependence or abuse is a common problem in patients, the need for more research examining the relationship with POD is mandatory. Other substance abuse is clearly associated with an increased risk of POD development [7,32]. Furthermore, as stated by Akira Kudoh et al. chronic benzodiazepine users are at a higher risk of developing POD.

Summarizing, we can say with certainty that older age (>70 years old), preoperative cognitive impairment, dementia, pre-op comorbidities, schizophrenia, PD, substance abuse, chronic benzodiazepines usage, OSA, TKA, fracture, and increased blood loss during surgery are risk factors for developing POD.

#### 3.1.4. Diagnosis

There are many diagnostic tools and methods used in clinical practice to diagnose delirium. The most popular is the confusion assessment method (CAM), followed by DSM criteria IV [60]. Other tools less frequently used are the DSM ΙΙΙ-revised, DSM V, delirium symptom interview (DSI), and the delirium rating scale-revised-98 (DRS-R-98).

The CAM instrument, which can be completed in less than 5 min, consists of the following four criteria: acute onset and fluctuating course, inattention, disorganized thinking, and altered level of consciousness. The CAM algorithm for the diagnosis of delirium requires the presence of both the first and the second criteria, and either the third or the fourth criterion. It has high sensitivity and specificity, and is also simple to use [61]. The diagnosis via DSM criteria requires a more thorough evaluation in most cases by a psychiatrist, thus is harder to use in daily clinical practice.

Considering the above, CAM is an easy-to-use tool for the diagnosis of POD and should be used in clinics providing care for elective TKA/THA patients. Using the other methods is more time consuming, and some require specialist training, making their everyday use impracticable.

#### 3.1.5. Pathophysiology and Biomarkers

There have been efforts to identify biomarkers in blood/CSF, or even ultrasound findings, to predict the proportion of the population at risk of POD. Given the consistent association between cognitive impairment and POD, biomarkers of Alzheimer’s disease (AD) have been investigated as predictors. The apolipoprotein (APOE) e4 allele, the main genetic risk factor for AD; cerebrospinal fluid (CSF); amyloid b 42 (Ab42); total tau (T-tau); and phospho-tau (P-tau) have been studied. Reviewing the literature showed that the level of APOE e4 allele does not correlate with POD [25,47], although there is a correlation between low CSF ab42 and POD [47]. Zhongcong Xie et al. examined whether the lower preoperative CSF–Aβ/Tau ratio was associated with higher incidence of POD. They divided the participants into quartiles according to the levels of the preoperative CSF–Aβ/Tau ratio, and compared the incidence of POD among these quartiles. Their results showed that more POD incidences occurred in the lowest quartile of CSF–Aβ/Tau ratio than in the rest of three quartiles [44]. Although their seems to be a relationship between the AD biomarkers and POD, more research is needed to determine the specifics of this relationship, and make the use of biomarkers in the clinical practice possible.

Τhe pathophysiology of POD is not fully understood. One theory suggests that proinflammatory cytokines secreted in the periphery due to surgical trauma interact with the neural tissue, causing neuroinflammation, which leads to the syndrome’s manifestation. This interaction can occur either directly via vagal afferents, or indirectly through transportation through the blood–brain barrier or the periventricular areas, where the blood–brain barrier is incomplete or absent. The cytokine signal transmission stimulates the microglia to produce inflammatory cytokines, which results in neuroinflammation. Through this mechanism, surgical trauma may cause POD; thus, modulators of inflammation might play a key role in the development of this condition [62,63]. In this context, the cholinergic anti-inflammatory pathway, in which acetylcholine (ach) acts as a modulator of the immune response and inhibits proinflammatory cytokine production in a dose-depended manner, might have a key role in the pathophysiology of POD. This led researchers to investigate components such as cholinesterase activity (the enzymes that deactivate ach), and their relationship with POD [28]. The studies reviewed showed lower plasma activity of acetylcholinesterase (AChE) and butyrylcholinesterase (BuChE), preoperatively, in the patients that developed POD than in patients without POD [28,64]. Additional to the cholinergic anti-inflammatory pathway, some researchers have also investigated a relationship between CRP and POD, although their studies showed no correlation [65,66]. The differences in pre-op and post-op albumin (Δalb) was also studied as a potential predictor for POD, with results suggesting a statistically significant correlation [17,23]. In addition, hip and knee arthroplasty surgeries are associated with the embolism of materials such as air, cement, and fat. Considering this, some researchers investigated a possible correlation between the number of emboli and POD, with their studies showing no statistically significant results [3,67].

Another theory suggests that patients with microstructural abnormalities are more prone to develop POD under the stress of surgery, due to brain homeostasis disturbance. Cavallari M. et al. studied the association of brain microstructural anomalies, assessed by MRI before surgery using the diffusion tensor imaging (DTI) technique, with postoperative delirium incidence and severity after non-cardiac surgery. Their findings showed a significant association of presurgical DTI irregularities in a variety of brain regions, such as the thalamus and hippocampus, with postoperative delirium incidence and severity. The regions involved may explain the neuropsychological manifestations that take place in POD [68].

Summarizing, although the pathophysiology of POD is not fully elucidated yet, research has been conducted to examine potential biomarkers that predict POD, focusing on the putative pathophysiological pathways. So far, there seems to be a correlation between the pre-op Ache/BuChE activity and POD. Furthermore, low CSF, Ab42, Ab42/tau, and Ab40/tau seem to predict POD, although more studies are required to clarify the relationship between these factors and POD to make their use in clinical practice possible. Lastly, some studies showed a significant correlation between Δalb and POD.

#### 3.1.6. Treatment

**Scheme 2:**
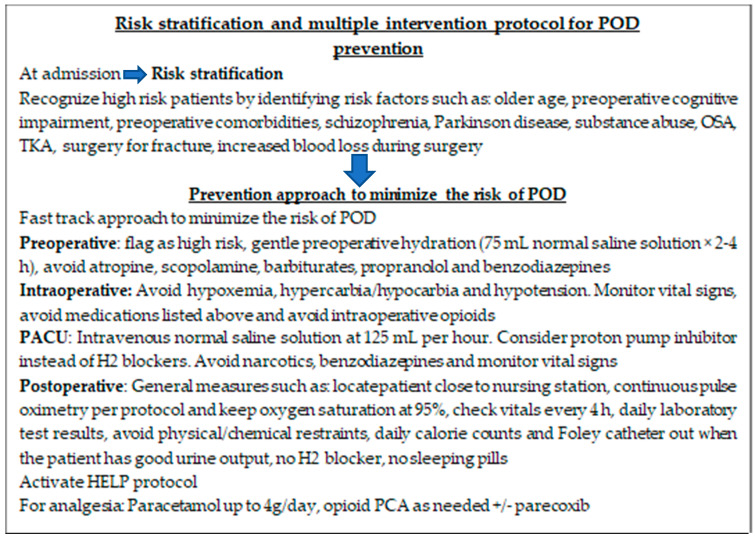
How to approach patient; risk stratification and multiple component prevention protocol for POD.

There is broad evidence that with the identification of patients at risk of developing POD and the initiation of a multiple component prevention program (Scheme 2), delirium is preventable in most cases [32,34,48]. Studies that implemented such protocols showed up to 99.33% POD reduction [48].

Protocols for risk stratification might include numerous risk factors, such as age, substance abuse, preoperative cognitive impairment (MMSE scores or other screening tools), sensory impairment, dependence in ≥1 ADL (activities of daily life), history of POD, history of falls, and chronic benzodiazepine usage [32,34,48]. A good example of a risk stratification protocol is the one used by Kristen E Radcliff et al., which is a score test including age, history of forgetfulness/hallucinations/falls/postoperative confusion, inability to perform higher brain functions; and performance on a simple mental exam. According to their score, patients are classified as low/medium/high risk [34]. After identifying the high-risk patients, a multiple component POD prevention approach should be set in motion, which includes preoperative, intraoperative, PACU (post anesthesia care unit), and postoperative evidence-based interventions.

Firstly, a fast-track approach should be used as it reduces the risk of POD [69].

Preoperative: Flag as high risk, gentle preoperative hydration (75 mL normal saline solution for 2–4 h) [32,34]. Avoid atropine, scopolamine, barbiturates, propranolol, and benzodiazepines [13,34].

Intraoperative: Avoid hypoxemia, hypercarbia/hypocarbia, and hypotension, and medications listed above [13,34]. Monitor vital signs, mode of anesthesia (regional or general) is not significant [11,16,21,40]. In addition, avoid intraoperative opioids [9,47,51].

PACU: Intravenous normal saline solution at 125 mL per hour. Consider proton pump inhibitor instead of H2 blockers. Avoid narcotics and benzodiazepines, and monitor vital signs [13,34].

Postoperative (general measures): Locate patient close to nursing station, continuous pulse oximetry per protocol and keep oxygen saturation at 95%, check vitals every 4 h, daily laboratory test results. daily calorie counts. Foley catheter out when the patient has good urine output. Avoid physical/chemical restraints; no H2 blocker, no sleeping pills [13,34].

For analgesia: Paracetamol up to 4 g per day, half lidocaine patch 5% for 12 h per day on each side of incision once surgical dressing is removed [34]. Opioid patient control analgesia (PCA) should be used if needed, since multiple studies showed no correlation with POD [18,49,52,70,71]. Furthermore, addition of parecoxib reduced the incidence of POD [24]. Some studies reported lower incidence of POD in patients that received nerve blocks in addition to PCA vs. patients that received PCA alone after the operation [36,72]. Some studies suggest that the use of olanzapine (10 mg) postoperative as a preventive measure for POD yields positive results and drastically reduces the incidence of POD [17,51].

In addition, according to the hospital elder life program (HELP) protocol: Orientation (reality/time), frequent communication with patient, involvement of family in daily care, one-to-one supervision to help with eating/toileting/turning, alternative methods to help with sleep, and quick mobilization of patient out of bed with physical therapy. These are measures that have to be applied in high risk individuals in order to prevent delirium [34].

### 3.2. POCD

POCD is defined as a syndrome of prolonged impairment of cognitive function, occurring weeks to months after surgery, and its diagnosis is possible by comparing the results of pre-op and post-op neurocognitive domain testing. Features of POCD include limitations in memory, intellectual ability, and executive function, and it is distinct from delirium and dementia [73]. The International Study of Postoperative Cognitive Dysfunction (ISPOCD) stated that patients had cognitive dysfunction when two Z-scores in individual cognitive tests, or the combined Z-scores, were 1.96 or more. This definition took into account general deterioration (in all tests) or substantial deterioration in only some tests, and it is applicable to all nationalities. The ISPOCD divides POCD as early (1 week post-op) and late (3 months post-op), whilst other studies also report a third category of one-year incidence of POCD [74,75,76,77,78]. It is important to clarify that early POCD is distinct from late POCD, and the presence of the one entity does not increase the risk of the other [65].

#### 3.2.1. Epidemiology

**Figure 1:**
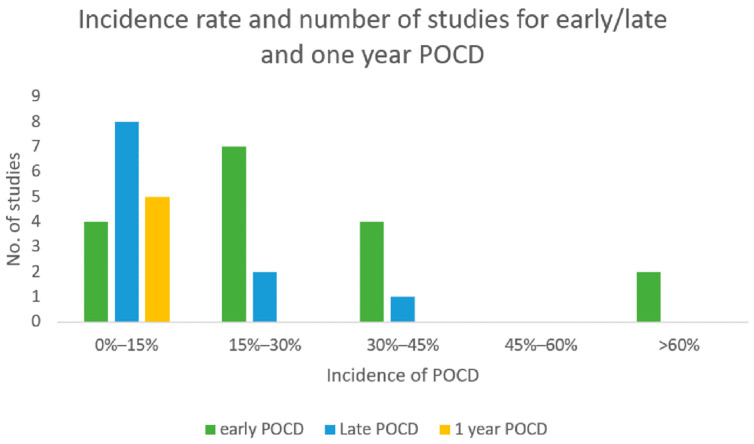
Incidence of early/late/1-year POCD and number of studies reporting it, respectively.

The literature shows a wide range of incidence for early and late POCD. More specifically, early POCD (6.7%–75%) with a median of 19.3%, and late POCD (8%–45%) with a median of 10% [65,66,69,75,76,77,78,79,80,81,82,83,84,85,86,87]. Some studies report the one-year incidence of POCD with a range of (0%–13.7%) and a median of 2.8% [75,76,77,78] (Figure 1). The high variation between studies can be attributed to the great variety of tests that are used to assess neurocognitive function.

#### 3.2.2. Risk Factors

Identifying risk factors for POCD after elective THA/TKA is of utmost importance, since the basis for treatment is the elimination of these factors, in addition to other interventions. Older age (>70 years, with a range of 65–82 years, as reported in various studies) and a high BMI seem to correlate with increased risk of POCD [84,85,86,88,89]. Studies that examined the relationship between sex and POCD show conflicting results, with Li Yan et al. stating that females are more prone to POCD, and Lene Krenk et al. stating that there is no relationship between sex and POCD [65,89]. Perhaps, the difference these in results is due to the fact that the studies examined different populations with different surgical protocols. For example, Li Yan et al. included patients >18 years old undergoing THA, while Lene Krenk et al. included patients >60 years old undergoing fast-track THA and TKA.

Preoperative cognitive impairment is associated with POCD, although we cannot be certain that this applies in both early and late POCD [65,76,89]. Since POCD is a condition defined by the impairment of neurocognitive domains, the need to investigate a possible relationship between cognitive reserve and POCD is obvious. The concept of cognitive reserve (CR) suggests that innate intelligence or aspects of life experience, such as educational or occupational attainments, may supply reserve in the form of a set of skills or repertoires that allows people to cope with cognitive impairment conditions better than others (i.e., progressive AD) [90]. Postler et al. found that a higher education level was associated with a lower incidence of POCD, and Si-Hai Zhu et al. stated that a lower education level was associated with a higher incidence of POCD. While these studies show a correlation between education level and POCD, J E Scott et al. stated that cognitive reserve in their study correlated with a specific test in a specific subset of patients; thus, they were not able to make a general statement on CR and POCD [86,88,91]. Summarizing, we can say that higher CR, and especially higher education level, acts as a protective factor to POCD.

Some studies examined the role of OSA and POCD, and showed no correlation [65]. Regarding mode of anesthesia (general vs. regional) or BIS (bispectral index) and POCD, no correlation was found [40,78,92,93,94,95,96]. In addition, active warming during surgery, cerebral oxygen desaturation, and uneven saturation during surgery, were shown to increase the incidence of POCD [85,87,97,98]. Si-Hai Zhu et al. showed that perioperative transfusion of >3 units of blood is associated with increased risk of POCD at 1 week; however, it is not clear if this is due to the underlining condition that mandated the need for transfusion or the proinflammatory effects from the transfusions themselves. The authors were unable to understand which scenario increased the risk because they did not measure the time course of the inflammatory mediators [88].

#### 3.2.3. Diagnosis

POCD diagnosis comes with its definition. It is achieved by comparing the preoperative and postoperative scores of different neurocognitive tests. The most frequently used test was the mini-mental state exam (MMSE), followed by the Montreal cognitive assessment (MoCA). Furthermore, there were many different variations of domain-specific tests (i.e., tests for memory, executive function, etc.) These tests are performed by giving the patient several cognitive tasks, such as finding similarities, naming objects, recalling words, and more, in order to assess several cognitive abilities.

As with POD, researchers attempted to identify biomarkers to predict POCD. Alzheimer’s biomarkers (CSF AB 42, P-tau, P-tau) were investigated as possible predictive markers for POCD. Reviewing the literature, we found that lower CSF, Ab42, and T-tau/Ab42, and P-tau/Ab42 are correlated with POCD incidence [28,75], while T-tau and P-tau alone do not correlate with POCD [66,75]. However, still more studies are needed to make these results clinically applicable. In some studies, the relationship between CRP and POCD was investigated without any correlation [65,66]. In addition, many studies investigated the relationship between the intraoperative emboli and POCD, and again found no correlation [67,79,80,99].

#### 3.2.4. Treatment

**Scheme 3:**
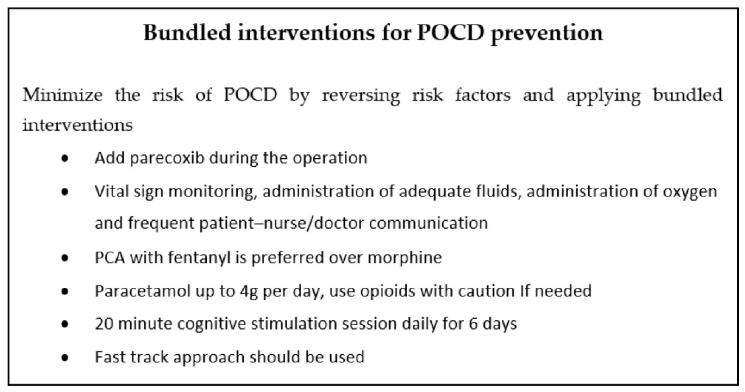
Bundled interventions for POCD risk reduction.

There is no specific treatment for POCD, and usually it resolves itself within a period of months. The solution for the clinician, to minimize POCD risk to their patient, is reversing the risk factors and applying some of the interventions described in the literature (Scheme 3 and Table 3).

General measures in order to ensure adequate brain oxygenation and keep the patient physiologically stable should be used. Vital sign monitoring, administration of adequate fluids to keep the patients hemodynamically stable, administration of oxygen, and frequent patient–nurse/doctor communication are such measures. In addition, a fast-track approach should be used [65,69], the mode of anesthesia not being significant [40,78,92,93,94,96]. For the pain, if needed, opioids can be used with caution and with appropriate doses, since they do not correlate with POCD [65,69]. PCA with fentanyl is preferred over morphine, since the latter was associated with lower MMSE scores [79]. Additionally, our literature review showed two more interventions that can be used. The first one is supported by a study conducted by Chia-Min Cheng et al., in which patients were submitted to a 20 min cognitive stimulation session daily for 6 days. This session included the recalling of past events, word games, and current event discussion. The intervention group showed lower POCD than the control group [100]. The second is the addition of parecoxib during the operation, which lowered early POCD, although it had no effect on late POCD [101].

## 4. Conclusions

POD after elective THA/TKA is a common complication that is related with increased morbidity. It has a median incidence of 14.8% and a high variation between different studies due to heterogeneous study protocols. Knowledge of the risk factors is mandatory since their implementation in risk stratification protocols is the basis of treatment. The diagnosis can be achieved with CAM, which is a tool that can be easily used in daily clinical practice. After risk stratification, multiple component prevention programs should be activated in high-risk patients. Regarding pain, the current literature review shows that opioid patient-controlled anesthesia does not correlate with POD, and, if needed, it should be used in order to relieve pain. In addition, adding parecoxib and/or nerve block to the PCA reduces the risk of POD. Future research should focus on clarifying the relationship between risk factors such as depression and alcohol dependence/abuse with POD, since they are both common diseases in the population. In addition, more research is needed in order to make the use of predicting biomarkers feasible in clinical practice.

POCD is a frequently occurring condition after elective THA/TKA, with an incidence of 19.3% for early and 10% for late POCD. Cognitive reserve and preoperative cognitive decline correlates with POCD. The diagnosis is made by comparing the scores of preoperative and postoperative neurocognitive tests. Previous research explored the relationship between Alzheimer’s markers preoperatively and POCD, with the results showing a correlation, although their use in clinical practice remains impracticable. More research is needed to make their use on a daily basis possible. While most patients recover in a period of months, specific interventions, in addition to general measures, are needed to reduce the risk of POCD. Future research should focus on quantifying the relationship between cognitive reserve and preoperative cognitive decline with POCD. Studies that examine the connection between preoperative cognitive test scores and POCD can lead to this quantification.

## Figures and Tables

**Table 1:** Incidence rate and No. of studies for POD.

Incidence Rate of POD	No. of Studies
0–5%	7
5–10%	3
10–15%	11
15–20%	2
20–25%	5
25–30%	3
>30%	4

**Table 2:** Summarized results for POD.

Risk factors	Diagnosis of POD	Treatment
Older age (>70) Pre-op comorbidities Pre-op cognitive impairment Dementia Schizophrenia Substance abuse Chronic benzodiazepines usage OSA PD	CAM4 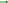 criteria: (1). Acute onset and fluctuating course (2). Inattention (3). Disorganized thinking (4). Altered level of consciousness Diagnosis require the first 2 criteria and one of the remaining two	Risk stratification and implementation of a multiple component prevention protocol for high risk patients

**Table 3:** POCD results summarized.

Risk Factors	Diagnosis	Treatment
Older age High BMI Preoperative cognitive impairment Perioperative transfusion of >3 units of blood Cerebral oxygen desaturation or uneven saturation during surgery	Comparing the preoperative and postoperative scores of different neurocognitive tests	Minimize the risk of POCD development by reversing risk factors and applying interventions Fast-track approach should be used Add parecoxib during the operation Vital sign monitoring Administration of adequate fluids Administration of oxygen Frequent patient–nurse/doctor communication PCA with fentanyl is preferred over morphine Paracetamol up to 4 g per day, use opioids with caution if needed 20 min cognitive stimulation session daily for 6 days

## Data Availability

Not applicable.
